# Developing an Interactive Environment through the Teaching of Mathematics with Small Robots †

**DOI:** 10.3390/s20071935

**Published:** 2020-03-30

**Authors:** Lilia Muñoz, Vladimir Villarreal, Itza Morales, Joseph Gonzalez, Mel Nielsen

**Affiliations:** 1 Grupo de Investigación en Tecnologías Computacionales Emergentes, Universidad Tecnológica de Panamá, El Dorado, Panama City 0819-07289, Panama; lilia.munoz@utp.ac.pa (L.M.); itza.morales1@utp.ac.pa (I.M.); joseph.gonzalez3@utp.ac.pa (J.G.); mel.nielsen@utp.ac.pa (M.N.); 2 Centro de Estudios Multidisciplinarios en Ciencias, Ingeniería y Tecnología-AIP (CEMCIT-AIP), Panama City 0819, Panama

**Keywords:** educational robotics, mathematics, primary education, teaching-learning

## Abstract

The article is the product of the study “Development of innovative resources to improve logical-mathematical skills in primary school, through educational robotics”, developed during the 2019 school year in three public schools in the province of Chiriquí, Republic of Panama. The teaching-learning process in students is influenced by aspects inside and outside the classroom, since not all schools have the necessary resources to deliver content or teaching material. The general objective of the project is to design, develop and implement educational robotics to improve logical-mathematical skills aimed at preschool and first grade students in public schools, using programmable educational robots. For this, a set of resources and activities were developed to improve the logical-mathematical skills of the initial stages, in public schools, obtaining significant results. Playful activities favor the teaching-learning process. Considering the analysis of the results made on the data obtained through the applied collection instruments, it can be argued that in general terms the values indicate that the students obtained a favorable level of performance in the different challenges proposed. The project has allowed the academic community to have an application of great value that allows teaching about the conservation of natural sites. The project only covers the area of mathematics in preschool and first grade.

## 1. Introduction

Technology in education has made great contributions in the dynamization of teaching methods applied to students, however, some experts believe that the pace at which these educational activities are transformed could improve, by what Magro said: “The change of education through technology is still a pending issue”. Digital technology has become an addition to education and not a priority given that in many educational centers the technology is not implemented due to geographical, economic or political factors that interfere with its acquisition and accessibility [1].

Certainly, technology affects the way of teaching and the key to its correct application is to change the traditional education paradigm: instead of students being taught to memorize content in a systematic and empty way, they should seek to develop their abilities and analysis skills, critical and logical thinking in their teaching regardless of the subject. The process of implementing technology in education not only involves its use, but also that the student is educated about its operation, possibilities of modification and creation of a new tool based on basic principles and concepts. To make this possible it is necessary to generate technological innovation projects that benefit schoolchildren. One of the tools used in education is educational robotics, but what is robotics? This is nothing more than the one in charge of studying the design and construction of machines or equipment capable of performing specific tasks [2].

Knowing this previous concept, it can be established that educational robotics involves the design and creation of teams (robots) that are developed with the purpose of educating students. Educational robotics has become a tool for teaching in the classroom providing more creative and adaptable methodologies to the different contents of education. It allows students to design, develop, modify, build solutions and analyze problems, taking advantage of the functionalities of existing robots, but applying them to different areas of primary education. Educational robotics tends to be also known as pedagogical robotics, since it consists in the conception, development and implementation of a robotic model or prototype, with the objective of using traditional teaching methodologies, evaluation rubrics and content taught in classes [3]. Now, despite the fact that educational robotics is known as pedagogical robotics, these differ in the following:

- Educational robotics makes use of robotics kits that include sensors, motors, plates and programmable kits (the acquisition of which is expensive in many cases), that is, the technological and electronic part is used more in teaching students.
- While pedagogical robotics integrate traditional knowledge in mathematics, technology and natural sciences, learning the basics of computer science without having a large amount of equipment, using low-cost technology.

Both make use of teaching methodologies that evaluate students to achieve the objectives indicated by assignment and through didactic guides a step-by-step follow-up of the task is given.

Instructing programming concepts to children from an early age, helps to them develop logical-mathematical skills that are beneficial when solving a problem and thus reduce the levels of failure that exist in the area of mathematics and content comprehension. There are a large number of robots used as teaching tools and for different educational levels, from simple robots for preschool children to later ages using increasingly advanced robot kits to develop students’ analytical abilities.; in such a way that when this child reaches a stage of secondary education and even university students, he can already carry out complex projects that involve the use of sensors, microcontrollers and multiple robotics kits to create solutions that benefit health, environment, education, construction of works civilians and other problems in their environment. 

There are many benefits that are obtained in the long term if from the initial stages of primary education students are taught the use of robotics and basic programming so that a better understanding of the contents given in the classroom is achieved [4].

The general objective of the project is to design, develop and implement educational robotics to improve logical-mathematical skills aimed at preschool and first grade students in public schools, using programmable educational robots.

The first grade and preschool students will be taken as a point of reference, integrating educational activities that will allow, on one hand, the achievement of curricular objectives in mathematics, as well as the development of the digital skills and competencies described above, as well as including in the process technology tools oriented to the programming of educational robots. For this, three public schools in the province of Chiriqui, Republic of Panama, have been selected. One of the schools is a multi-grade school in the rural area, and the other two belong to the urban area.

The article is structured as follows: Section 2 presents an overview of robotics in teaching. Section 3 shows the methodology used, while Section 4 presents the results and discussion and Section 5 describes some conclusions and future work.

## 2. Robotics in Teaching-Learning

Robotics in education has been implemented in different countries of Europe and America as mentioned in [4,5,6,7,8,9,10,11], among others, making the use of educational robotics more and more popular inside and outside the curricula of different educational centers around the world.

Through the educational robots, students can enter this new technological world, and, in addition, they are one of the best didactic tools for the teaching of the Science, Technology, Engineering and Mathematics (STEM) academic disciplines. In this sense, different researchers have shown that the interaction of students with programmable educational robots, as is the case of the Bee-Bot in appropriate educational contexts, have promoted the acquisition of mathematical and geometric concepts in a significant way [12,13,14], as well as obtaining several achievements acquired by students through experimentation with the Bee-Bot, and the application of different strategies to discover their functions and characteristics [15,16,17].

For their part, Angeli and Vanadines presented in their work [18] a study that examined the effects of learning with the Bee-Bot on young boys’ and girls’ computational thinking within the context of two scaffolding techniques. The study reports statistically significant learning gains between the initial and final assessment of children’s computational thinking skills. Also, according to the findings, while both boys and girls benefited from the scaffolding techniques, a statistically significant interaction effect was detected between gender and scaffolding strategy showing that boys benefited more from the individualistic, kinesthetic, spatially-oriented, and manipulative-based activity with the cards, while girls benefited more from the collaborative writing activity.

On the other hand, Greenberg et al. [19] showed how students can be guided to integrate elementary mathematical analyses with motion planning for typical educational robots. Rather than using calculus as in comprehensive works on motion planning, they show students can achieve interesting results using just simple linear regression tools and trigonometric analyses. Experiments with one robotics platform showed that use of these tools can lead to passable navigation through dead reckoning even if students have limited experience with the use of sensors, programming, and mathematics.

Another interesting work is the one developed by Estivill-Castro [20] where the author developed three lessons supported by the principles of inquiry-based learning (IBL) and problem-based learning (PBL) in educational robotics with the aim of steering and emphasizing the mathematics aspects of the curriculum and the role of mathematics in STEM, while also touching on the social context and impact of STEM.

The learning of mathematics represent, together with reading and writing, one of the fundamental learnings of elementary education, given the instrumental nature of these contents. Hence, understanding the difficulties in learning mathematics has become a manifest concern of many of the professionals dedicated to the world of education, especially if we consider the high percentage of failure in these contents presented by students who complete the compulsory schooling. In this scenario, the learning of this subject involves complex processes that require a great diversity of methodologies to achieve the maximum possible efficiency. The use of ICT and educational robotics is especially well suited to this matter: the use of images, graphs, spreadsheets, etc. in calculators and computers it allows us to advance very quickly and, most importantly, to understand and retain the necessary information. In addition, these tools open the possibility of creating new learning environments and, therefore, of developing new methodologies that make the most of the resources available. The work context addressed by the development of this project is mathematics in initial education, using for this gamification through educational robots, with the aim of strengthening the logical-mathematical skills in early stages.

## 3. Methodology

The project will develop a set of innovative resources for teachers and students of first grade and preschool level in public primary schools, to improve the teaching-learning process of mathematics, using programmable educational robots as a low-cost robotic element. The methods and materials that will be used are described below. 

### 3.1. Sample

This study was carried out with a sample of 240 students in kindergarten and first grade of primary education (from 4 to 7 years old) to evaluate the use of the Bee-Bot robot as a tool for the teaching-learning process of mathematics. None of the students had used this tool before, making it the first time they have faced this type of methodology in the classroom. The project has the approval of three (3) schools from different locations, two of them in urban areas and one in a rural area. One of the schools is multigrade. All schools are public schools.

### 3.2. Methods

The project includes two stages, the first with a theoretical basis, which contemplates a systematic review of the literature of the subject under study. In addition, a set of recreational activities organized in didactic guides for teachers and students will be developed in this stage. These will be accompanied by a set of rubrics, checklists and questionnaires that will allow the resulting data to be collected. The second stage is of an experimental type through training sessions for teachers and students who participate, based on the area of mathematics; all this with the support of programming tools and educational robotics appropriate to the educational level.

**Figure 1:**
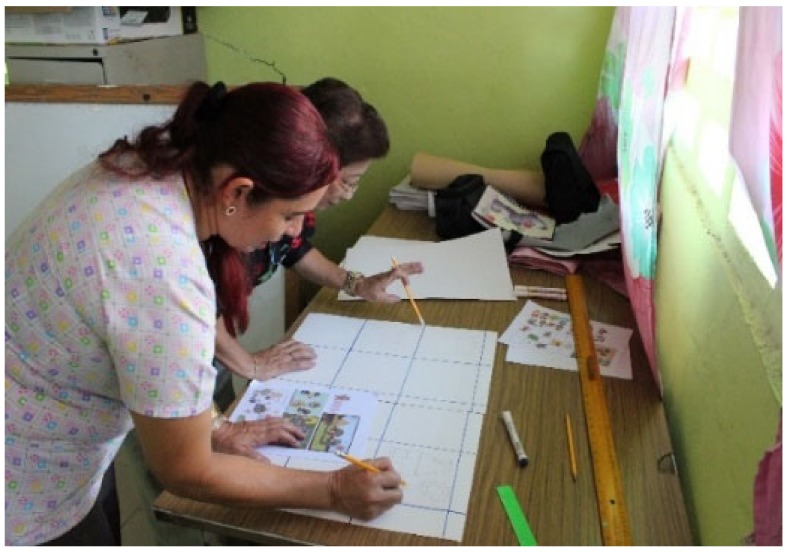
Practical workshop for making rugs.

**Figure 2:**
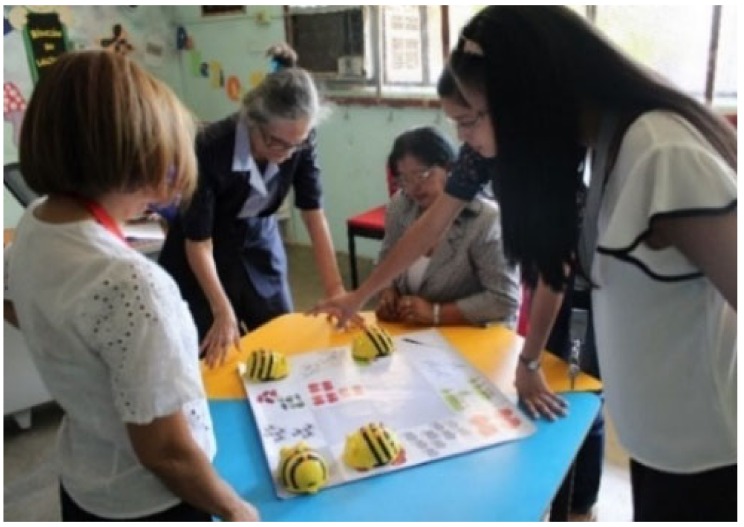
Practical workshop on the use of Bee-Bot.

An important aspect that was considered for the successful development of the project was the training of the teachers who participated in the project. Figure 1 shows part of the training for teachers, where they are being instructed in the creation of rugs. Figure 2 shows teachers using Bee-Bots.

The training of the children was given within the activities of the project. This activity consisted of a weekly visit to the schools. In collaboration with the teachers, visiting hours were established, each session lasted one hour. In the first sessions the students were instructed in the use of Bee-Bot, the use of the buttons, how it was turned on, turned off and how the instructions were erased. Later he began working with the guides, and in each session, they were developing the planned activities.

**Figure 3:**
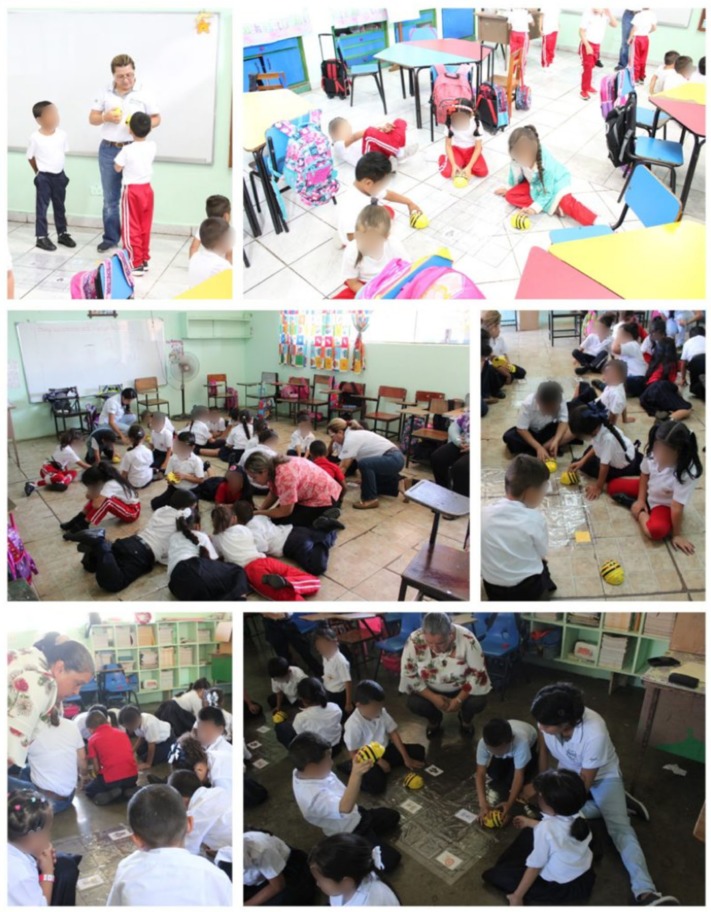
Practical workshop on the use of Bee-Bot with students.

It is important to mention that previously the teachers were consulted if the topic we were going to develop had already been given in class, this allowed to carry out the activities in a correct way, in turn allowed to reinforce what the teacher had previously explained. The implementation of the different activities in the classrooms have been developed in accordance with the school curriculum of the kindergarten and first grade levels. Next, in the Figure 3 one can see images of the activities developed in each of the participating schools.

**Figure 4:**
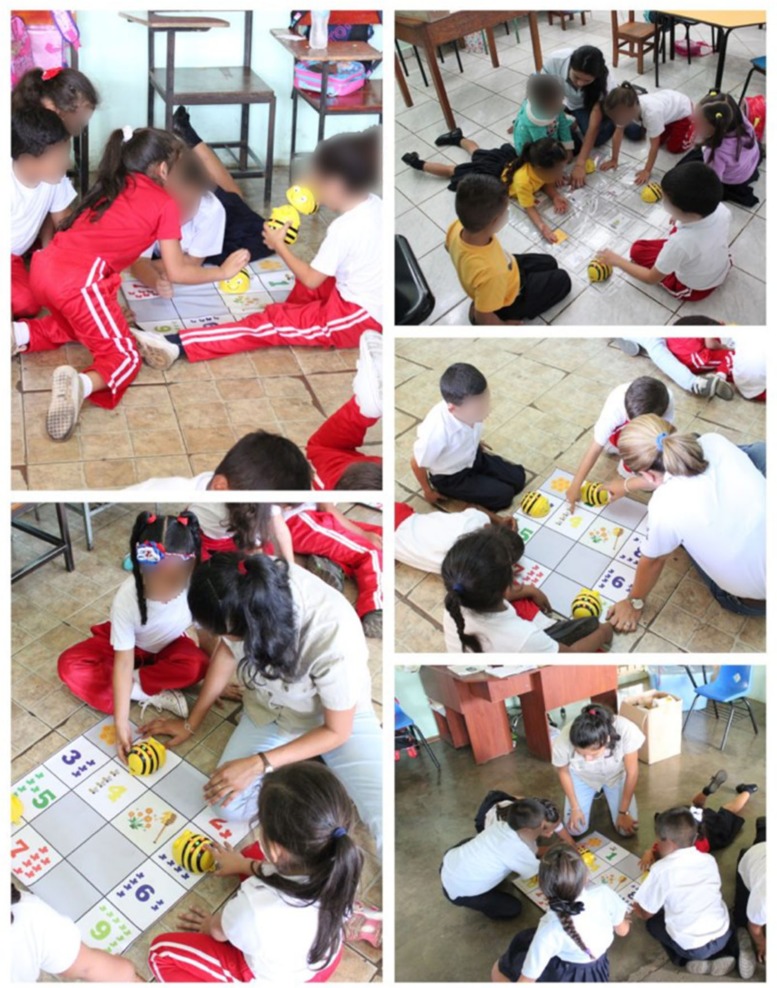
Activities developed by students designed by teachers and researchers.

After learning how Bee-Bot worked, different activities were planned for each level. This activity was divided by academic level. Figure 4 shows some of these activities.

- Kindergarten:
  The activity consisted of students deducing the necessary instructions to make Bee-Bots travel until they arrive at the letter with the illustration studied with the help of the instructor in turn. The objective of this activity was to strengthen the concepts of spatial location with terms such as inside–outside, above–below and in front–behind, while at the same time reinforcing to students the correct way to make Bee-Bots work.
- First grade:
  On the other hand, in this second activity, first grade students were reminded of the numbers they saw the previous year when they were in kindergarten as stipulated in the school curriculum.

The activity was carried out with the help of a grid mat in which some cells presented the symbolic representation and in number of objects of the numbers, so that the students were able to count the spaces that Bee-Bot had to travel to reach the number indicated by the instructor, promoting the association of the name of the number with its symbology and equivalent quantity.

### 3.3. Materials

**Figure 5:**
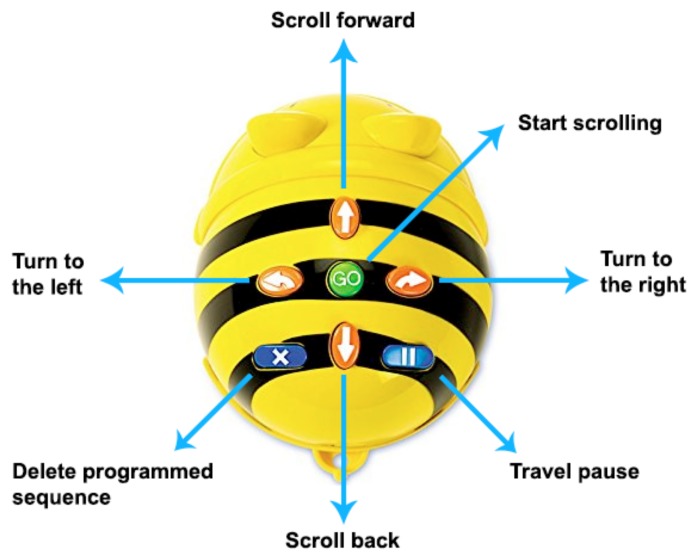
Bee-Bot® Robot: description of buttons and their functions. Source: Own elaboration.

As a tool for programmable educational robotics activities, the Bee-Bot Kit [21] was used, which is an educational material designed to develop the elementary capacities of programming and computational thinking, such as: spatial location and cognition, motor skills and perception, logic and strategy. These robots perform movements at 90° angles and must be programmed to follow a coherent sequence on each mat, so with proper programming, the robot bee will be able to find the answers to an addition to give an example, each time it stops in a space, depending on the mat that is used with the kit. The equipment that will be used in the schools has been acquired, as it can be seen in Figure 5. Internally the robot can store up to 40 instructions in its memory. The movements will be executed sequentially. The displacements allow the robot to travel with a fixed distance of 15 cm forward or backward. The left or right turns you can make correspond to an angle of 90º. Once the robot carries out the programmed sequence of movements, a sound is produced, and the robot’s eyes light up indicating to the participant that the execution of the movement program has been carried out.

**Figure 6:**
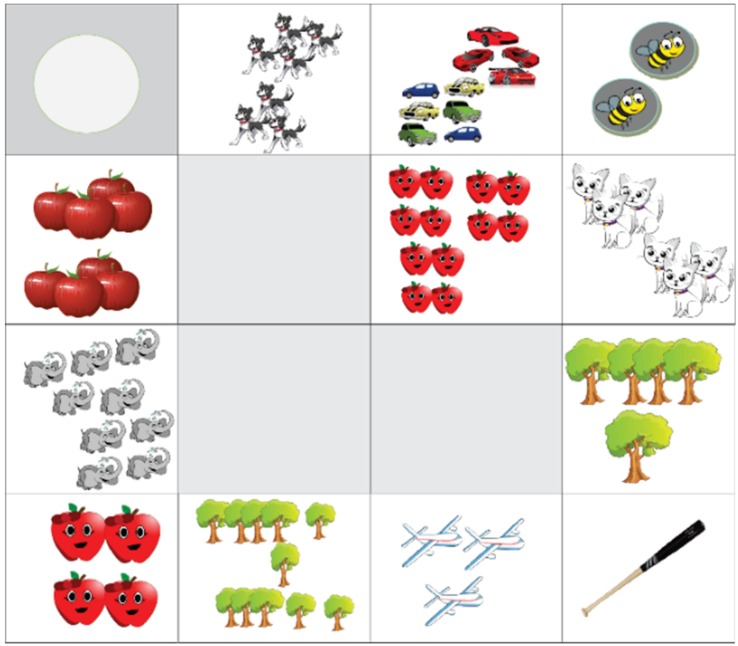
Mat to learn to count directed toward children at preschool level. Source: Own elaboration.

Besides, computers will be used for development of activities in the classroom as well as mobile devices such as tablets and smartphones to perform the tests. Some mats have been produced to develop the first activities. Figure 6 shows one which sought to familiarize preschool children with numbers from one to twelve; for this purpose, images that can attract the attention of children have been used. A scheme has been developed for the execution of every activities; an example can be found in Table 1. 

For the development of the project, meetings were held with authorities and directors of the Ministry of Education (MEDUCA from for its initials in Spanish), with the purpose of presenting the project and at the same time obtaining the permits to entry the schools where the project will be executed.

### 3.4. Design and Varibles

We propose a pre-experimental design, since its degree of control is minimal and, in this way, we can obtain information on the phenomenon under study. In this case, the information will be obtained at the beginning and at the end of the project. For this scenario, questionnaires were developed to collect information on previously established variables. 

The variables used to display the results are the following:

- Robotics resources: It is capable of autonomously using robotics materials in the classroom.
- Programming sequence: The student has command of programming commands and is able to establish a correct sequence.
- Learning by inquiry: It can overcome small challenges through robotics and programming. Observing and analyzing, although it is difficult for him to reflect on his own mistakes.
- Building solutions: Shows interest in the functioning of objects. Investigate and try to build with the proposed materials a mechanism to solve a problem, with a purpose.
- Teamwork: Accept help without interruption, collaborate with peers.

### 3.5. Data Collection Tools

To evaluate the performance of each student in the different challenges proposed, a rubric was prepared. The rubric used was an adaptation of the “SSS rubric” instrument developed by the DevTech research group and implemented in the TangibleK robotics study program [22,23].

The criteria and values used in the evaluation rubric were aimed at measuring student performance in the design and construction of the sequence of movements that responded to the proposed challenge.

Variables were established to evaluate the activities. Table 2 shows the rubric. For this, a pre-test and a post-test were planned. The variables that were established have allowed to validate several aspects of the teaching-learning process of the project. The criteria and values used in the evaluation rubric were aimed at measuring student performance in the design and construction of the sequence of movements that responded to the proposed challenge.

The pre-test was carried out in the second week of training, after having explained to the students the operation of the Bee-Bot robot and having worked with them on an activity. The post-test was carried out in week 10 of the training, the students, in this scenario, had already developed skills in handling Bee-Bot and had advanced in the content of the subject.

For the experimental design, parents were given a document on informed consent and all parents approved and accepted that their children participate in the experimental stage of the project.

### 3.6. Data Analysis

The analysis of the data obtained through rubric was based on a descriptive study of the data reported by the different items in which the percentage of responses of the scale (one to five) for each variable was calculated. Data were presented through Excel charts, with which the analysis of the data obtained could be performed. A comparison of the means of the pre-test and post-test was carried out. The *t*-student was used with the statistical package SPSS version 23 (IBM, New York, NY, USA).

## 4. Results and Discussion

The evaluation of the activities was carried out jointly by professors and researchers. The activity started with a short narrative. Then the problem or challenge to be solved was raised. For example, moving the Bee-Bot^®^ robot to a specific position. The exact location where the robot was to move was presented in the initial narrative. This type of challenge is associated with the sequences dimension as part of the computational thinking skills explored in the experience.

### 4.1. Graphs and Analysis of the Data Obtained

The analysis of the data obtained in the pre-test and post-test for each of the established variables is presented below.

#### 4.1.1. Robotic Resources 

We evaluated whether the student was able to autonomously use robotics materials in the classroom, for which he was previously given instructions on how Bee-Bot works.

##### Kindergarden

**Figure 7:**
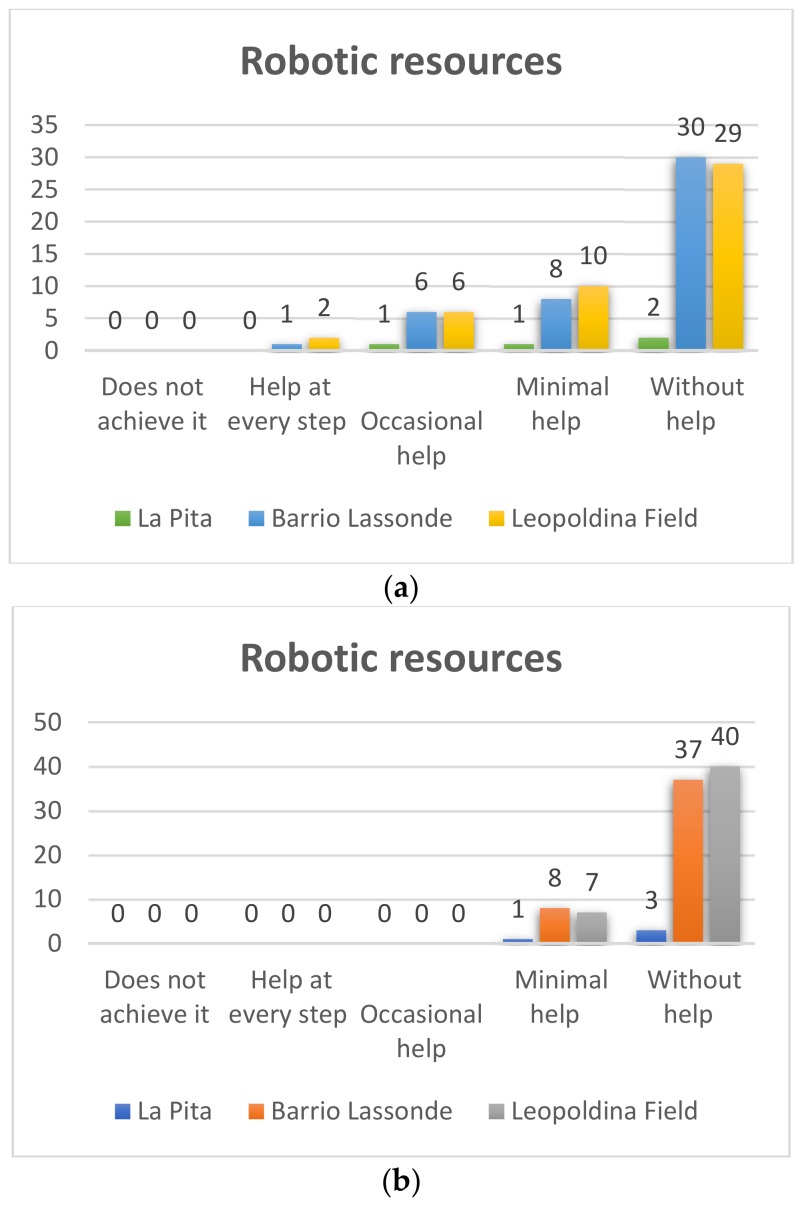
Kindergarden robotic resources Pre-Test (**a**) and Post-Test (**b**).

- Pre-Test
  The pre-test was developed in order to evaluate the performance of students in the use of Bee-Bot in the early stages. The results of each of the evaluated variables are presented below. In Figure 7a it can be seen that in Leopoldina Field School of the 47 students who participated in the project 29, which represents 62% of the total number of students, used the Bee-Bot robots without help and only 10 students (21%) needed minimum help to use the robot, while six (13%) needed occasional help and one student (4%) needed help in each execution step. On the other hand, as for the students of Barrio Lassonde School, 30 of the 45 students managed to use the Bee-Bot robot without help, which represents 67% and eight students (18%) managed to use the robot with minimal help; 6 students (13%) occasionally needed help, and one student (2%) needed help with each step. For its part, at La Pita School, of the four students who participated in the project two students managed to use the Bee-Bot robot without help, which represents 50%, one of the students achieved it with minimal help (25%) and one required occasional help (25%).
- Post-Test
  The results of the post-test can be seen in Figure 7b, where it is observed that in the Leopoldina Field School of the 47 students who participated in the project 40, which represents 85% of the number of students, used the Bee-Bot robots without help and only seven students (15%) learned to manage to use the robot with minimum help. On the other hand, as for the students of Barrio Lassonde School, 37 of the 45 students (82%) managed without help to use the Bee-Bot robots, and eight students representing 18% managed to use the robot with minimal help. At La Pita School of the four students who participated in the project, three (75%) managed to use the Bee-Bot robots without help, and one of the students (25%) achieved this with minimum help. With these results one can see the significant progress that students have had in the use of robotic resources.

##### First Grade

**Figure 8:**
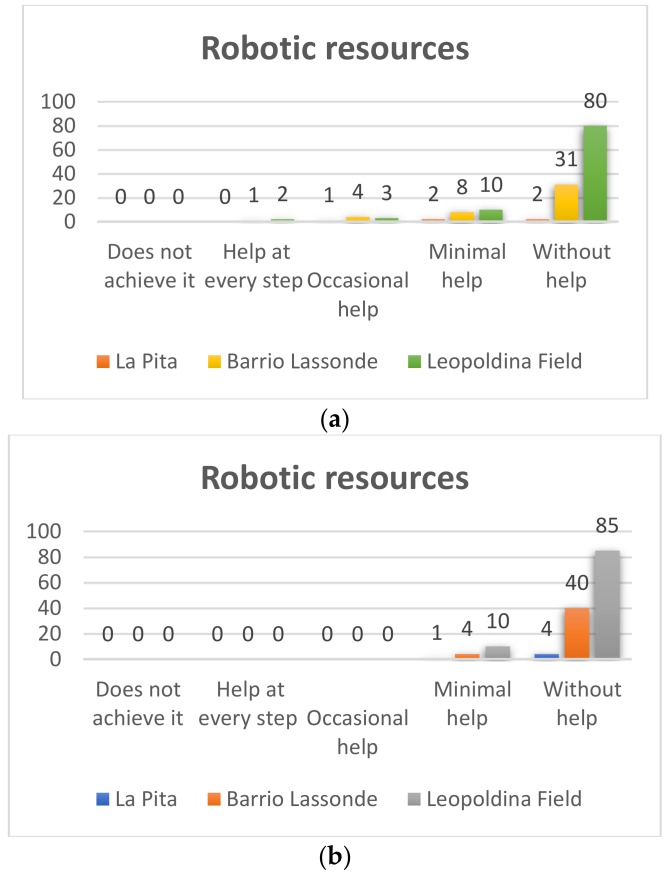
First grade robotic resources Pre-Test (**a**) and Post-Test (**b**).

- Pre-Test
  In the first grade, this variable was also evaluated, and the results were as follows. In Figure 8a it can be seen that in the Leopoldina Field School of the 95 students who participated in the project 80 (84% of the total), used the Bee-Bots without help and only 10 students (11%) needed minimum help to use the robots, while three (3%) required occasional help, and two representing 2%, needed help for each step. On the other hand, regarding the students of Barrio Lassonde School, 31 of the 44 students (71%) managed to use a Bee-Bot robot without help, and eight students representing 18% managed to use the robot with minimal help, while four students (9%) achieved it with occasional help, and we found one student (2%) who needed help at each step. For its part, at La Pita School of the five students who participated in the project two students (40%) managed to use the Bee-Bot robots without help, and two of the students required minimal help (40%) and while 1 (20%) achieved it with occasional help.
- Post-Test
  In the post-test the results shown in Figure 8b, it can be observed that in the Leopoldina Field School, 85 of the 95 students who participated in the project (89%) used the Bee-Bot robots without help and only 10 students (11%) needed minimum help to managed to use the robots. On the other hand, of the students of Barrio Lassonde School, 40 of the 44 students (90%) managed to use the Bee-Bot robots without help, and four students representing 1% managed to use the robot with minimal help.At La Pita School of the five students who participated in the project four (80%) managed to use the Bee-Bot robots without help, and only one of the students (20%) needed minimum help,. These results show a significant advance of students in the use of robots.

#### 4.1.2. Programming Sequence

In this aspect it was sought that the students recognize an incorrect sequence, at the time of programming, and also if he/she mastered the programming commands and if he/she was able to establish a correct sequence. The results of this variable are shown below.

##### Kindergarden

**Figure 9:**
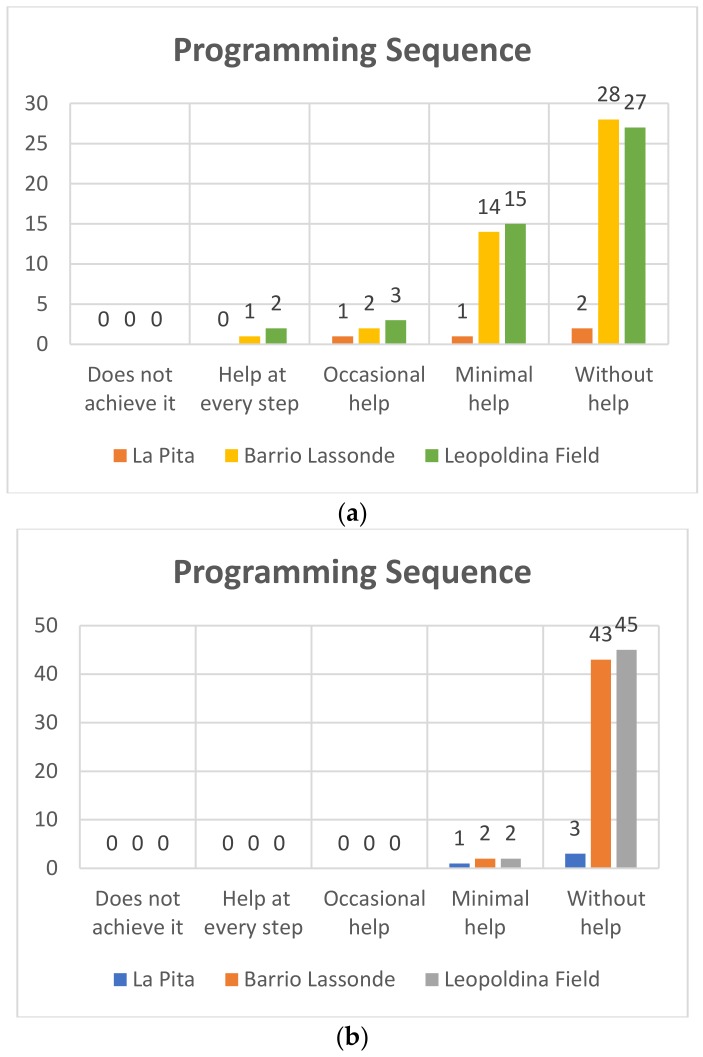
Kindergarden programming sequence Pre-Test (**a**) and Post-Test (**b**).

- Pre-Test
  In Figure 9a it can be seen that of the 47 students who participated at Leopoldina Field School, 27 (58%) managed to establish a correct programming sequence without help, 15 students achieved it with minimal help (32%), while three students (6%) needed occasional help, and two students (4%) needed help at each step. On the other hand, at the Barrio Lassonde School, 28 of the 45 students (62%) managed to carry out a correct sequence without help, while 14 students (31%) needed a minimum of help to complete the task, two students (4%) required occasional help and one student, representing 2%, needed help at each step. At La Pita School the following results were obtained: of the four students who participated, two (50%) did not require help to achieve the programming sequences, one student (25%) needed minimal help, and one student (25%) achieved it with occasional help.
- Post-Test
  In Figure 9b, of the 47 students at the Leopoldina Field School who participated, 45 (95%) were able to establish a correct programming sequence without help, and two students achieved it with minimal help (5%). On the other hand, at the Barrio Lassonde School 43 of the 45 students (95%) managed to carry out a correct sequence without help, and two students (5%) needed a minimum of help to achieve it. The following results were obtained at La Pita School: of the four students who participated three (75%) did not require help to achieve the programming sequences, and one student (25%) needed minimum help. The progress that has been achieved in terms of programming sequence can thus be evidenced in the results of the post-test.

##### First Grade

**Figure 10:**
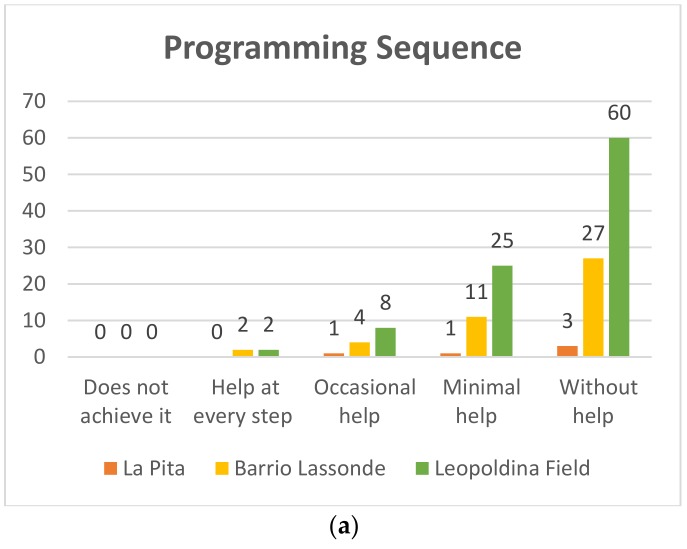

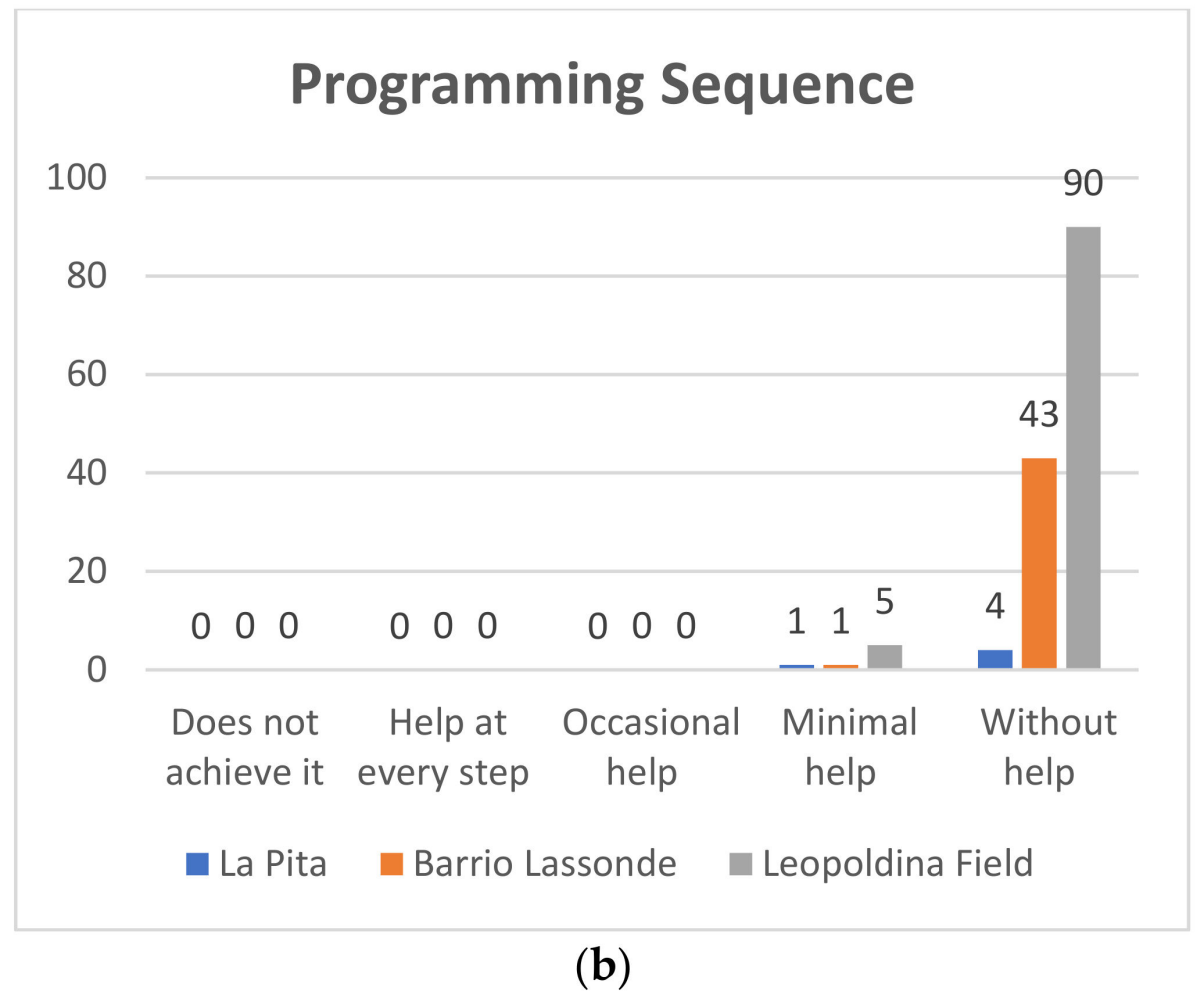
First grade programming sequence Pre-Test (**a**) and Post-Test (**b**).

- Pre-Test
  In Figure 10a it can be seen that at the Leopoldina Field School 60 of the 95 students who participated in the project, which represents 63%, used the Bee-Bot robots without help, whereas 25 students (26%) managed to use the robotd with minimum help, eight (8%) with occasional help, and two needed help in each step to achieve it, representing 2%. On the other hand, as for the students of Barrio Lassonde School, 27 of the 44 students (61%) managed to use using the Bee-Bot robots without help, and 11 students representing 25% managed to use the robot with minimal help, while four students achieved it with occasional help (9%) and we found that two students (5%) required help at each step. For its part, at La Pita School, of the five students who participated in the project three (60%) managed to use the Bee-Bot robots without help, and only one of the students (20%) achieved it with minimal help, while one achieved it with occasional help (20%).
- Post-Test
  In Figure 10b it can be seen that at the Leopoldina Field School 90 of the 95 students who participated in the project (95%) completed the task without help using the Bee-Bot robots and five students (5%) managed to use the robot with the least help. On the other hand, as for the students of Barrio Lassonde School 43 of the 44 students (98%) managed to use the Bee-Bot robots without help, and one student (2%) could use the robots with minimal help. For its part, at La Pita School, of the five students who participated three (75%) used the Bee-Bot robots without help, and only one of the students (25%) needed minimum help, When you compare the results of the pre-test and post-test you can see great progress.

#### 4.1.3. Inquiry Learning

With this variable, we sought to assess whether the students were able to overcome small challenges through robotics and programming by observing and analyzing, although it will be difficult for him/her to reflect on his/her own mistakes.

##### Kindergarden

**Figure 11:**
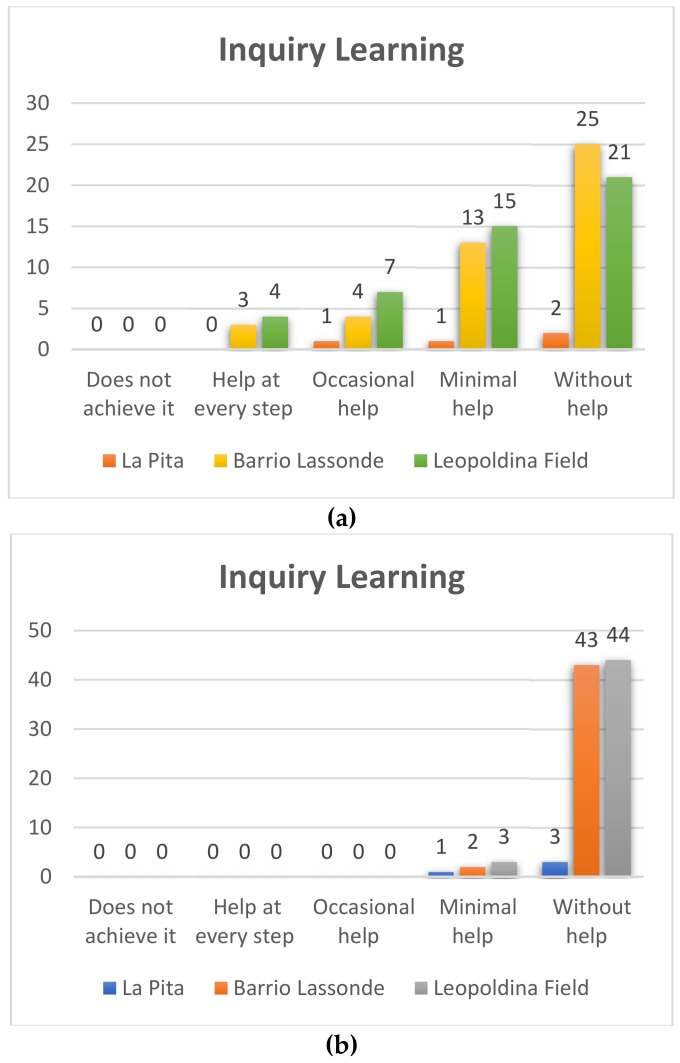
Kindergarden inquiry learning Pre-Test (**a**) and Post-Test (**b**).

- Pre-Test
  In Figure 11a it can be seen that at Leopoldina Field School, of the 47 students who participated 21 (45%) were able to establish a correct programming sequence without help, 15 (32%) of the students achieved it with minimal help, while seven students (15%) needed occasional help, and four students (8%) needed help at each step. On the other hand, at the Barrio Lassonde School, 25 of the 45 students (55%) managed to carry out a correct sequence without help, while 13 students (29%) needed a minimum of help to achieve this, four students (9%) required occasional help and three students (7%) needed help at each step. The following results were obtained at La Pita School: of the four students who participated two (50%) did not require help to achieve the programming sequences, one student (25%) needed minimal help, and a student (25%) achieved it with occasional help.
- Post-Test
  In Figure 11b of the 47 students who participated at the Leopoldina School Field 44 (93%) managed without help to establish a correct programming sequence, and three students (7%) achieved it with minimal help. On the other hand, at the Barrio Lassonde School 43 of the 45 students (95%) managed to carry out a correct sequence without help, and two students (5%) needed a minimum of help. At La Pita School the following results were obtained: of the four students who participated three (75%) did not require help to achieve the programming sequences and one student (25%) needed minimal help. Very significant improvement results can thus be evidenced.

##### First Grade

**Figure 12:**
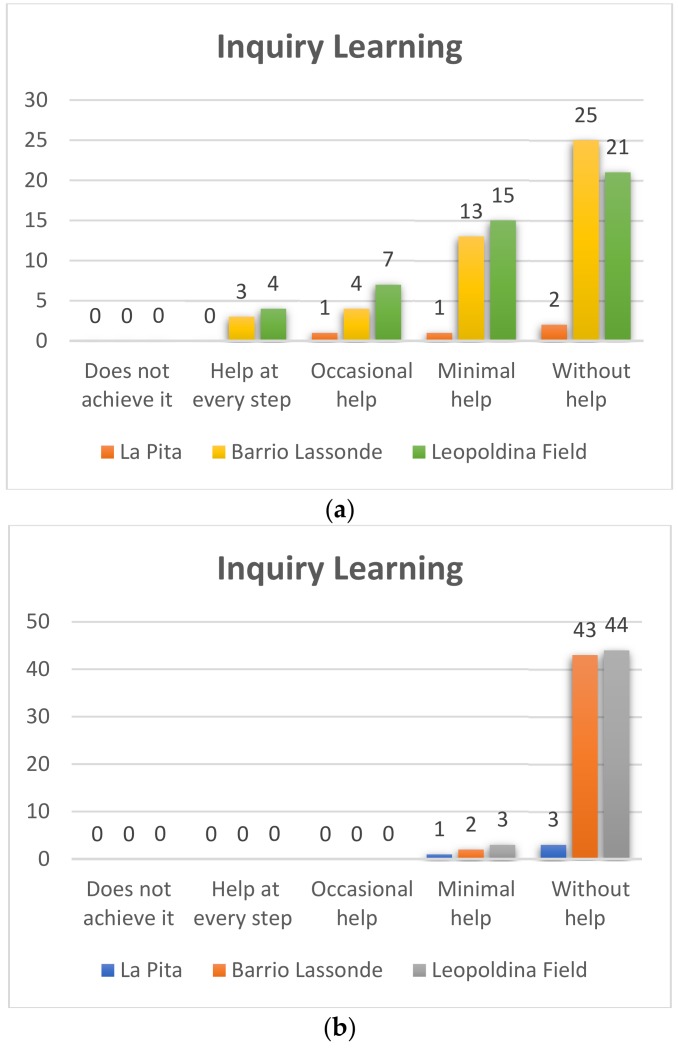
First grade Inquiry Learning Pre-Test (**a**) – Post-Test (**b**).

- Pre-Test
  Figure 12a shows the results of the Leopoldina Field School of the 95 students who participated in the 55 project, which represents 58% of the number of students without help using the Bee-Bot robot, 25 students representing 26% who they manage to use the robot with the minimum help, while 10 with occasional help, which represents 11% and 5 needed help at each step to achieve it, representing 5%.
  On the other hand, as for the students of Barrio Lassonde School, 22 of the 44 students managed to use the Bee-Bot robot without assistance, which represents 50% and 12 students representing 27% managed to use the robot with minimal help, while that 6 students achieved it with occasional help representing 13%, with help at each step we find 4 students equivalent to 9%.
  For its part, the La Pita School of the 5 students who participated in the project 3 students managed to use the Bee-Bot robot without help, which represents 60% and only 1 of the students achieves it with a minimum of help, representing 20%, while 1 achieves it with occasional help representing 20%.
- Post-Test
  Figure 12b shows the results of the Leopoldina Field School of the 95 students who participated in the project 92, which represents 97% of the number of students without help using the Bee-Bot robot and 3 students representing 3% They managed to use the robot with the least help.
  As for the students of Barrio Lassonde School, 43 of the 44 students managed to use the Bee-Bot robot without help, which represents 98% and 1 students representing 2% managed to use the robot with minimal help.
  On the other hand, in the La Pita School of the 5 students who participated in the project 4 students managed to use the Bee-Bot robot without help, which represents 80% and only 1 of the students achieved it with minimum help, representing 20%. When you buy the results of the pre-test and post-test you can see great progress.

#### 4.1.4. Building Solutions

Through this variable we evaluated whether the students showed interest in the operation of the objects, if they investigated and tried to build with the proposed materials a mechanism to solve a problem, it was also assessed if they worked with the purpose to reach the goal of the activity presented. The results are shown below.

##### Kinder Garden

**Figure 13:**
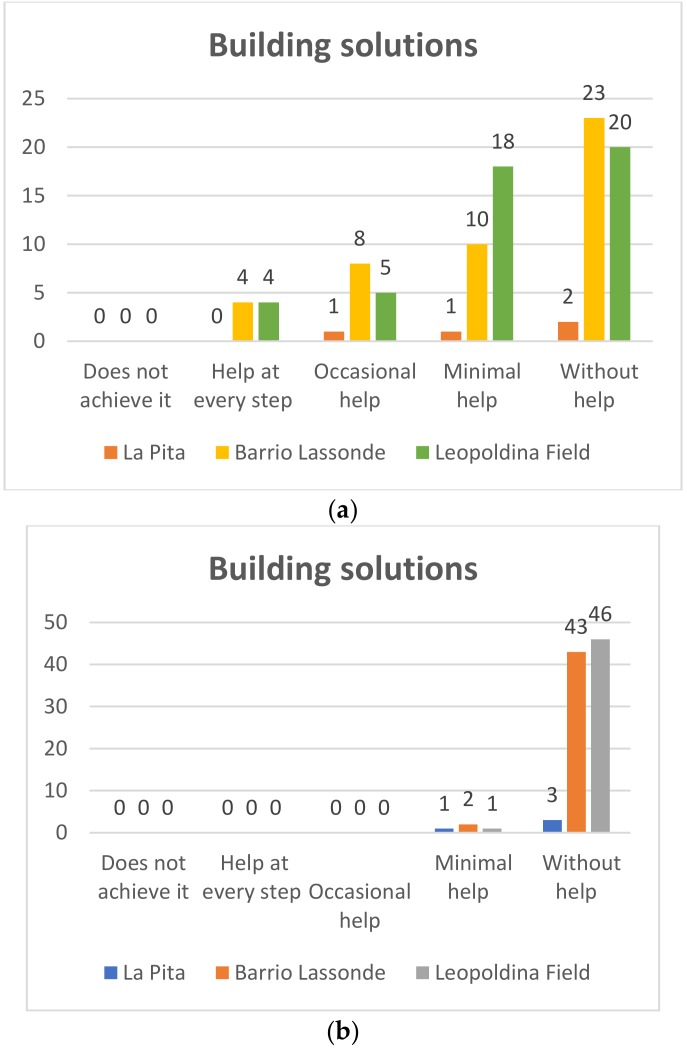
Kindergarden building solutions: Pre-Test (**a**) and Post-Test (**b**).

- Pre-Test
  In Figure 13a it can be seen that at the Leopoldina Field School, of the 47 students who participated in the project 23 (51%) managed to use the Bee-Bot robots without help and only 10 students (22%) who managed to use the robot needed the minimum help, while eight (18%) needed occasional help, and four students (9%) need help in each execution step. On the other hand, regarding the students of Barrio Lassonde School, 30 of the 45 students (67%) managed to use the Bee-Bot robots without help, and eight students (18%) managed to use the robots with minimal help, while six students (13%) occasionally needed help, and only one student (2%) needed help with each step. For its part, at La Pita School, of the four students who participated in the project two (50%) students managed to use the Bee-Bot robots without help, one of the students (25%) achieved it with minimum help, and one (25%) required occasional help.
- Post-Test
  In Figure 13b it can be seen that at the Leopoldina Field School of the 47 students who participated in the project 46 (98% of the total) could use the Bee-Bot robots without help and only one student (22%) managed to use the robot with the least help. On the other hand, regarding the students at Barrio Lassonde School, 43 of the 45 students (95%) managed to use the Bee-Bot robots, with no help and two students (5%) managed to use the robots with minimal help. For its part, at La Pita School of the four students who participated in the project three (75%) managed to use the Bee-Bot robots without help, and one of the students achieved it with minimum help (25%).

##### First Grade

**Figure 14:**
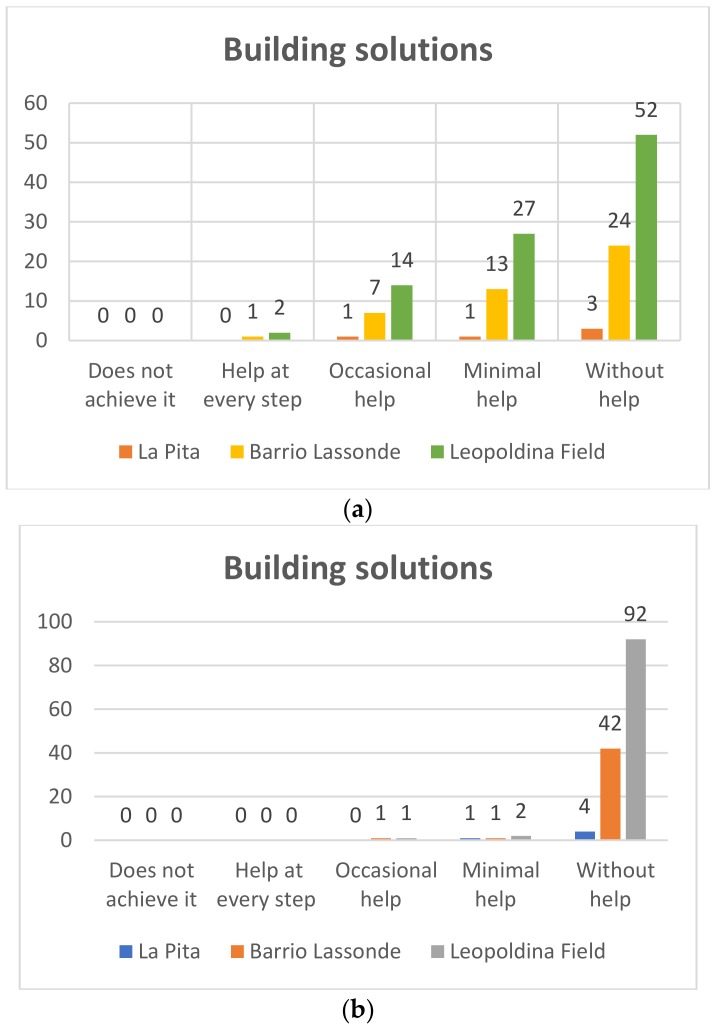
First grade building solutions: Pre-Test (**a**) and Post-Test (**b**).

- Pre-Test
  In Figure 14a it can be seen that at the Leopoldina Field School, of the 95 students who participated in the project, 52 (55%) used the Bee-Bot robots without help, whereas 27 students (28%) managed to use the robots with the minimum help, while 14 (15%) needed occasional help, and two (2%) needed help in each step. On the other hand, as for the students of Barrio Lassonde School, 24/44 students (54%) managed without help to use the Bee-Bot robotd, and 13 students (29%) managed to do so with minimal help, while seven students (15%) achieved it with occasional help and one student (2%) needed help at each step. For its part, at La Pita School, of the five students who participated in the project three (60%) could use the Bee-Bot robots without help, only one of the students (20%) achieved it with a minimum of help, and one (20%) achieved it with occasional help.
- Post-Test
  In Figure 14b it can be seen that at the Leopoldina Field School, of the 95 students who participated in the project 92 (97%) used the Bee-Bot robots without help, while two students (2%) required minimum help, and one student needed occasional help (1%). On the other hand, as for the students of Barrio Lassonde School, 42 of the 44 students (96%) managed to use the Bee-Bot robots without help, one student (2%) did do with minimal help and one student (2%) needed occasional help. For its part, at La Pita School, of the five students who participated in the project four (80%) managed to use the Bee-Bot robots without help, and only 1 of the students (20%) needed minimal help.

#### 4.1.5. Teamwork

With this variable, it was intended to evaluate the concept of teamwork, in this sense it was assessed if the students accepted contributions from their peers at the time of performing any of the scheduled activities. The results are shown below.

##### Kindergarden

**Figure 15:**
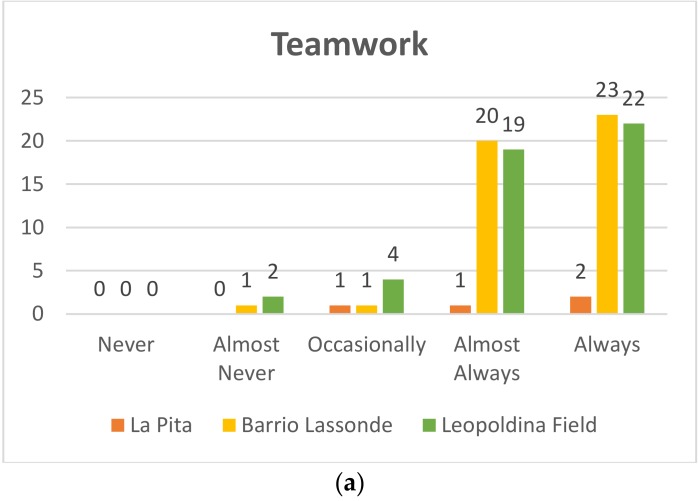

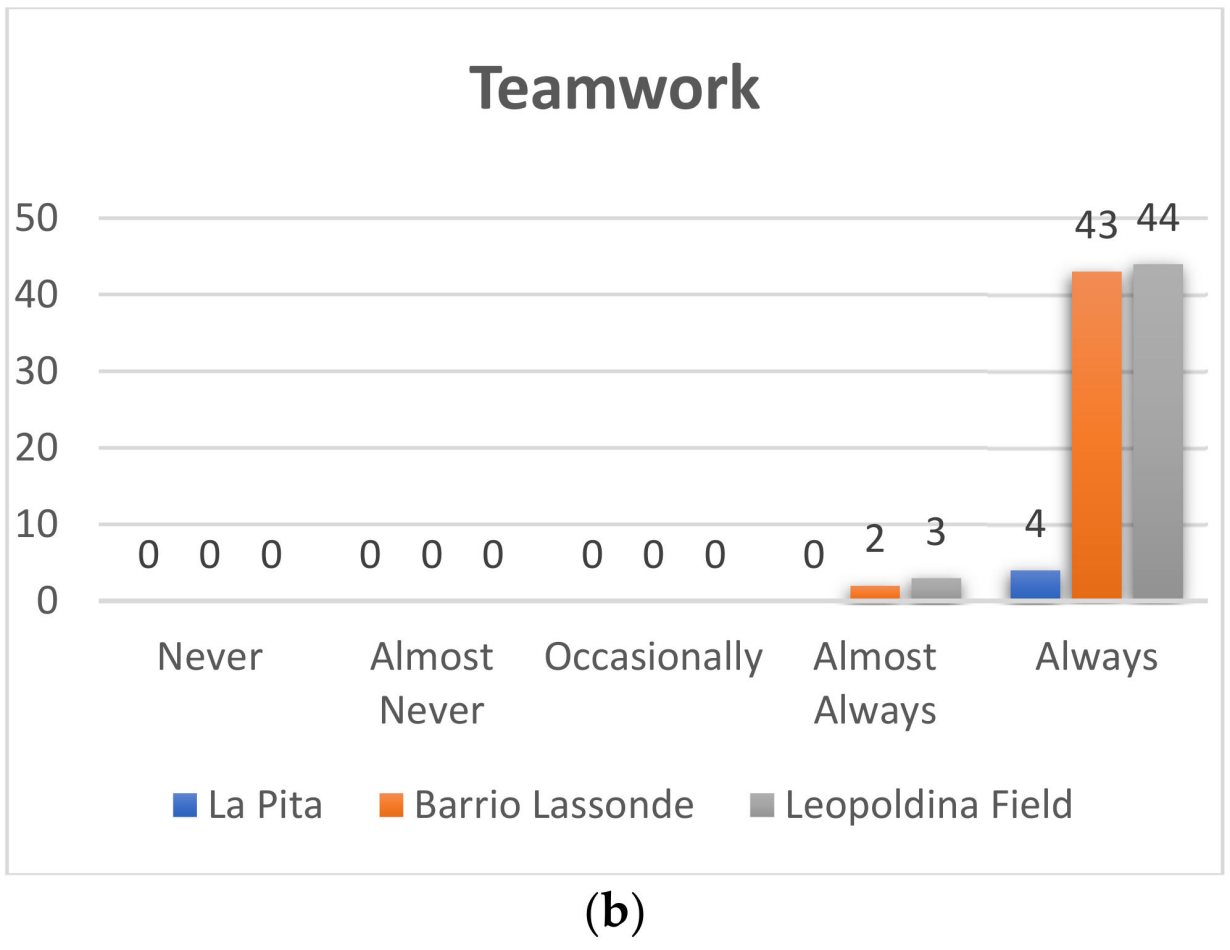
Kindergarden teamwork: Pre-Test (**a**) and Post-Test (**b**).

- Pre-Test
  In Figure 15a it can be seen that of the 47 students who participated at Leopoldina Field School, 22 (47%) managed to work in a team, 19 students (40%) almost always managed to work in a team, while four students (9%) occasionally worked in teams and two (4%) students almost never worked as a team. On the other hand, at Barrio Lassonde School, 23 of the 45 students (51%) achieved teamwork, 20 students (445) almost always worked in teams, one student (2%) occasionally worked in teams, and one student (2%) almost never worked as a team. The following results were obtained at La Pita School: of the four students who participated, two worked as a team (50%), one student (25%) almost always worked as a team, and one student (25%) occasionally worked as a team.
- Post-Test
  In Figure 15b of the 47 students who participated at the Leopoldina School Field 44 (93%) managed to work as a team, and three students (7%) almost always managed to work as a team. On the other hand, at Barrio Lassonde School 43 of the 45 students (95%) achieved teamwork, and the other two students (5%) almost always worked in teams. At La Pita School, 100% of the kindergarten students worked as a team.

##### First Grade

**Figure 16:**
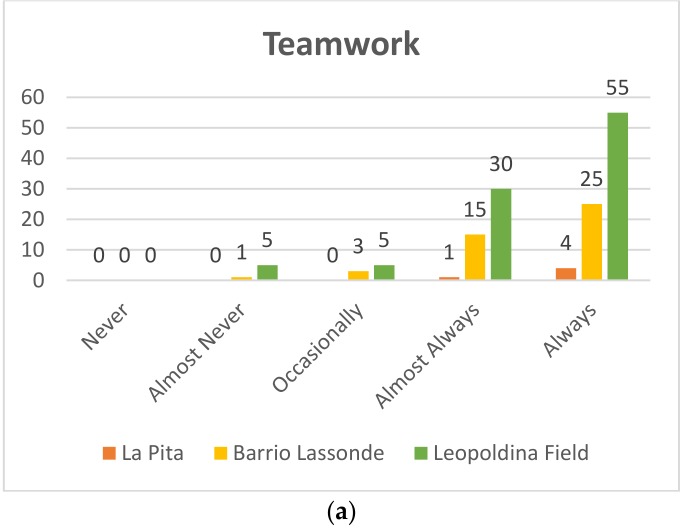

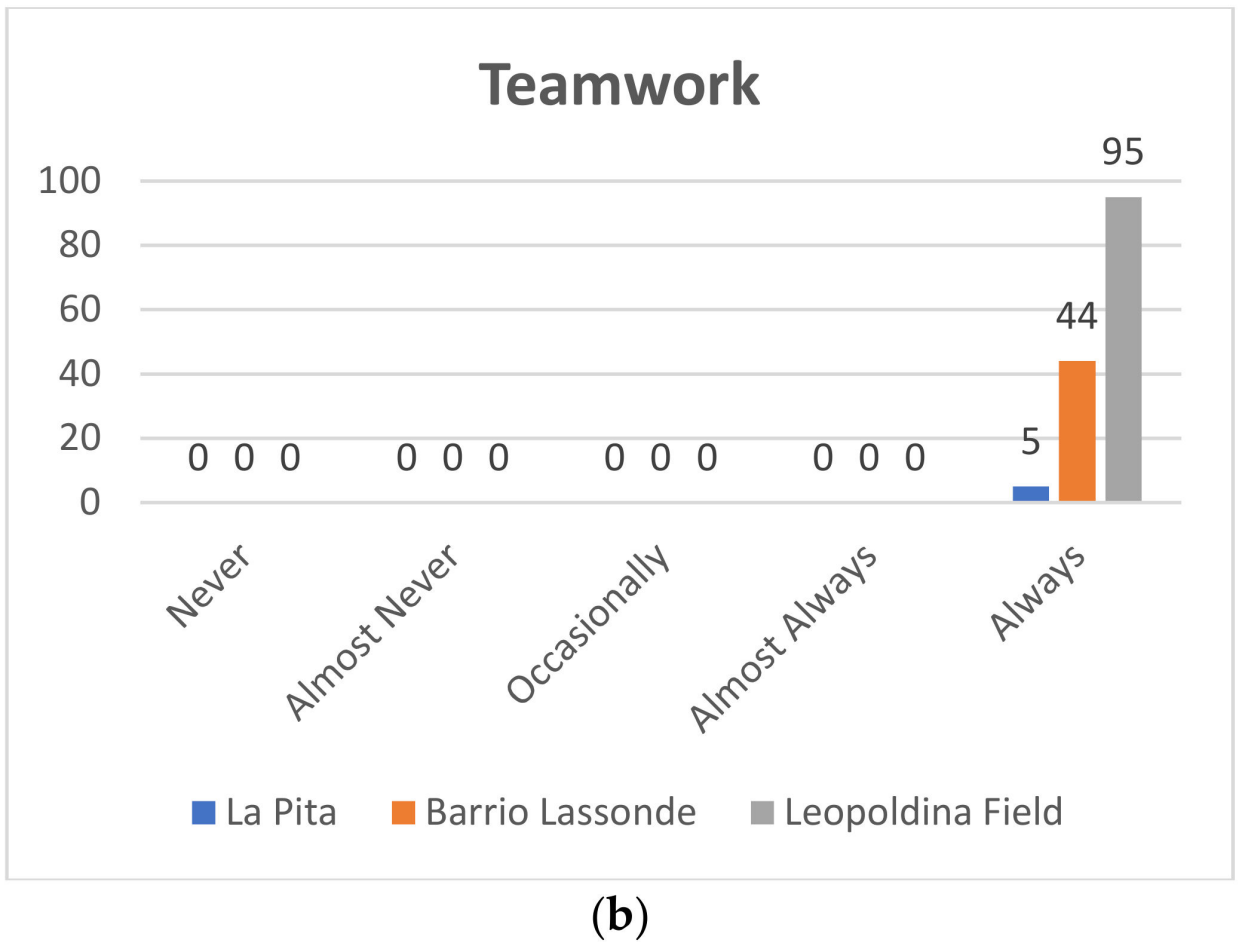
First grade teamwork: Pre-Test (**a**) and Post-Test (**b**).

- Pre-Test
  As seen in Figure 16a of the 95 students who participated at Leopoldina Field School 55 (58%) always worked as a team and 30 students (32%) almost always managed to work as a team, while five students occasionally worked as a team (5%) and another five students (5%) almost never worked as a team. On the other hand, at Barrio Lassonde School, 25 of the 44 students (57%) achieved teamwork, 15 students (34%) almost always worked in teams, three students (6%) occasionally worked in teams, while a student (2%) almost never worked as a team. The following results were obtained at La Pita School: of the four students who participated, three worked as a team (80%) and one student almost always worked as a team, which represents 20%.
- Post-Test
  In all the schools that participated in the project, students were able to work as a team, as can be seen in the Figure 16b.

### 4.2. Statistical Analysis

#### 4.2.1. Kindergarden

A *t*-student test was carried out for two related samples, in this sense, the pre-test and post-test results were taken. Table 3 presents the descriptive statistics generated from the data collected in both tests. It is observed that the calculated mean for the data collected in the post-test is higher than the mean of the pre-test. 

Table 4 shows the results of applying the statistical *t*-student test for samples related to the pre-test and post-test data. The results indicate significant differences in the calculated values that represent the performance of the students in the development of the proposed activities. As can be seen regarding the asymptotic significance (Sig.), Values lower than the reference value of *p* < 0.05 were obtained.

On the other hand, the effect size was calculated using Cohen’s d, the value obtained was d = 0.517. According to the theory [24], for this type of tests they are classified with the scale: small = 0.20; medium = 0.50; large = 0.80. In this case the effect is medium according to the results obtained. According to the data obtained, it can be affirmed that better results were obtained in the post-test evaluations.

#### 4.2.2. First Grade

As for kindergarden, a *t*-student test was performed for two related samples, in this sense, the pre-test and post-test results were taken. Table 5 presents the descriptive statistics generated from the data collected in both tests. It is observed that the calculated mean for the data collected in the post-test is greater than the pre-test means.

Table 6 shows the results of applying the statistical *t*-student test for samples related to the pre-test and post-test data. The results indicate significant differences in the calculated values that represent the performance of the students in the development of the proposed activities. As can be seen regarding the asymptotic significance (Sig.), Values lower than the reference value of *p* < 0.05 were obtained. On the other hand, the effect size was calculated using Cohen’s d, the value obtained was d = 0.595. According to the theory [24], for this type of tests they are classified with the scale: small = 0.20; medium = 0.50; large = 0.80. In this case the effect is medium according to the results obtained. According to the data obtained, it can be affirmed that better results were obtained in the post-test evaluations.

## 5. Conclusions and Future Works

In the first instance and in accordance with the general objective of the project, a set of innovative resources have been obtained for the improvement of logical-mathematical skills aimed at preschool and first grade students using programmable educational robots.

Considering the analysis of the data results obtained through the collection instruments applied, we can argue that in general terms the values indicate that the students obtained a favorable level of performance in the different challenges proposed. There is also a positive attitude and acceptance of the robotics resource and the learning activities carried out in teachers and students.

The development of the play and programming experience with the Bee-Bot® robots was effective and useful to strengthen the design, structure and evaluation mechanisms of planned activities.

On the other hand, it is important to contemplate in this article what is related to the size of the sample used, which due to limitations associated with the number of schools that allowed the development of the activity, could not be greater. In reference to this point, limits should be considered, for researchers, associated with policies and standards of each educational center in relation to the development of experimental studies. Therefore, these first results achieved represent a good starting point for the successful consolidation of the study.

Finally, the pre-test and post-test evaluations, using the sample of students who participated in the project, allowed us to value the activities and correct some previously raised. This has made it possible to strengthen the framework of scientific knowledge that exists in relation to the development of programming skills and computational thinking in early educational stages.

As future work, this project is intended to be implemented in other public educational centers in the province of Chiriquí, in addition to developing other work scenarios, with new activities for other areas or teaching subjects such as Spanish, Science, Arts or English.

## Figures and Tables

**Table 1:** Activity scheme.

Activity Name: Learning Numbers from 0 to 10 with Bee-Bot	
Objectives	To identify and memorize the numbers with the help of a Bee-Bot. Match the number of objects with the number for better compression. Speed up your logical capacity, so the Bee-Bot travels to the number indicated by the teacher.
Material Needed	Mat or number template Dimensions: 80 × 80 cm Bee-Bot
Recommended Age	4–6 years
Period	15–30 min
Competencies worked	Development of logical thinking, communication and collaboration. Maths Learning to learn Basic concept of displacement or trajectory and proximity of the number to be found. Spatial relations (right, left, forward, back).
Development of Activity	1) Form groups of three students to develop collaboration and communication skills. 2) Place the mat or templates on the floor and explain how they should perform the activity of the route with the numbers as well as the relationship between the number of objects. 3) Give the Bee-Bots to the students, so that they begin the journey; each time it reaches its final position, explain to the student the relation between object and number; this way they will easily memorize the numbers from 0 to 10. 4) At the end of the activity, the Bee-Bot must return to their origin/start point or to the cell of the flower with the honey according to the position closest to where the Bee-Bot is.
Complementary Activities	Form two teams of five students where they select three equal colors and find the fastest route, mentioning at the end of the ascending order of the numbers with the selected color.

**Table 2:** Rubric for first grade.

Assessment	Description
5 points	Understanding and complete achievement of the challenge, without the teacher’s help.
4 points	Understanding and significant achievement of the challenge, with minimal interventions or teacher aids.
3 points	Understanding and satisfactory achievement of the challenge, receiving help from the teacher throughout the process, but not step by step.
2 points	Understanding and achieving the challenge with step-by-step help from the teacher.
1 point	The solution to the challenge was initiated, but it was not completed.

**Table 3:** Descriptive statistics for the pre-test and post-test values in Kindergarten.

	N	Mean	Standard Deviation	Standard Error Mean
Pre-Test	96	3.177	1.8732	0.1912
Post-Test	96	4.167	2.4192	0.2469

**Table 4:** *t*-student test for related samples of pre-test and post-test data in Kindergarden.

	Mean	Standard Deviation	Standard Error Mean	95% Confidence Interval of the Difference		*t*	gl	Sig.
				Lower	Upper			
Pre-Test Post-Test	–0.9896	2.0026	0.2044	–1.3953	–0.5838	–4.842	95	0.000

**Table 5:** Descriptive statistics for the pre-test and post-test values in First Garde.

	N	Mean	Standard Deviation	Standard Error Mean
Pre-Test	144	3.92	1.533	0.128
Post-Test	144	4.48	2.062	0.172

**Table 6:** *t*-student test for related samples of pre-test and post-test data in First Grade.

	Mean	Standard Deviation	Standard Error Mean	95% Confidence Interval of the Difference		*t*	gl	Sig.
				Lower	Upper			
Pre-Test Post-Test	–0.556	1.577	0.131	–0.815	–0.296	–4.228	143	0.000
